# Mindfulness and false memories: state and dispositional mindfulness does not increase false memories for naturalistic scenes presented in a virtual environment

**DOI:** 10.1007/s00426-021-01504-7

**Published:** 2021-04-13

**Authors:** Julia Ayache, Kouloud Abichou, Valentina La Corte, Pascale Piolino, Marco Sperduti

**Affiliations:** 1 Laboratoire Mémoire, Cerveau and Cognition (LMC2 URP 7536), Institut de Psychologie, Université de Paris, 71 Ave Édouard Vaillant, Ile de France, 92100 Boulogne-Billancourt, France; 2 Department of Psychology, Nottingham Trent University, Nottingham, United Kingdom; 3 Institut de la Mémoire et de la Maladie d’Alzheimer (IM2A), Département de Neurologie, Hôpital Pitié-Salpêtrière, AP-HP, Paris, France; 4 Institut Universitaire de France, Paris, France

## Abstract

Mindfulness attracted increased research interests in the last decade, reporting an overall beneficial effect of this practice on cognitive performances. Nevertheless, recently a possible detrimental impact of mindfulness has been underlined. While the effect of mindfulness on memory remains under-explored, recent studies have observed an increased false-memory susceptibility after mindfulness practice. A possible explanatory mechanism has been suggested, related to the nature of the studied material. For semantically related information, mindfulness would increase false memories; however, the addition of rich perceptual information could prevent this detrimental effect. The present study aimed to verify this hypothesis by testing the impact of state mindfulness induced by a short meditation session, and dispositional mindfulness on the production of false memory for pictorial material presented in a complex virtual environment. We employed a virtual reality version of the Deese–Roediger–McDermott paradigm (DRM), a classical protocol to induce false memories. Contrary to previous studies, we did not observe any effect of mindfulness on false or correct memories (free recall and recognition) after a short mindfulness practice session compared to a control condition. Nonetheless, we found a beneficial effect of mindfulness practice on memory sensitivity. Additionally, we reported a positive and negative effect of dispositional mindfulness on memory outcomes. While the Non-Reactivity facet was associated with overall better memory performances, we observed an association between the Acting with Awareness facet and an increased recollection of lures. We discuss these findings in line with a recent proposal on the link between mindfulness and episodic memory.

**Supplementary Information:**

The online version contains supplementary material available at 10.1007/s00426-021-01504-7.

## Introduction

Since its introduction in medical practice by the pioneering work of Kabat-Zinn, mindfulness has received increasing attention from the scientific community. Several results highlighted the benefits of this practice on psychological well-being and cognitive performances, particularly on attentional and executive functions (Chiesa et al., 2011). Nevertheless, recent studies reported some unexpected side-effects, observing an increase of false memories after mindfulness practice (Rosenstreich, 2016; Wilson et al., 2015). More generally, the impact of mindfulness on memory processes has received few attentions, and results remain controversial.

Defined as “paying attention in a particular way: on purpose, in the present moment, and non-judgmentally” (Kabat-Zinn, 1994, p. 4), mindfulness is a specific mental state, cultivated by the practice of meditation but also considered as a personality-like trait beyond any meditation training (Baer et al., 2006). Therefore, we will employ mindfulness meditation and dispositional mindfulness to indicate the former and the latter. Several cognitive models have described mindfulness principally as an attentional control training (Lutz et al., 2008; Tang et al., 2015). Indeed, a wide range of studies have pointed out the effect of short- and long-term practice on different attentional components (Jha et al., 2007; Schofield et al., 2015; Tang et al., 2007). Given the established link between attention and memory (e.g., Naveh-Benjamin et al., 2000; Sperduti et al., 2017a), one should expect a beneficial effect of such practice on memory processes. Furthermore, memory is a central concept within the traditional Buddhist conception of mindfulness. One of the meanings of the word “sati” (the original *pali* world translated with mindfulness) is “mindful and able to recollect and remember what has been done or said long ago” (Anālayo, 2016, p. 3). Surprisingly the effect of mindfulness meditation on memory has received little attention until recently.

### Mindfulness and memory

Growing scientific evidence casts a new light on the complicated relationship between mindfulness and different memory systems (Levi & Rosenstreich, 2019). For example, a study comparing long-term mindfulness practitioners to demographically matched controls reported better performances for short- and long-term memories for the mindfulness practitioners (Lykins et al., 2012). Nevertheless, the results reported by studies investigating the impact of short meditation practices and dispositional mindfulness on autobiographical and episodic memory reveal a more contrasting picture. For instance, different studies reported a reduction of autobiographical memories over-generalization, a tendency usually observed in clinical depression (Hermans et al., 2008), after a mindfulness intervention in both clinical (Hargus et al., 2010) and non-clinical populations (Heeren et al., 2009; Williams et al., 2000). On the contrary, Crawley (2015) observed the opposite effect for dispositional mindfulness, with a decreased autobiographical memory specificity. The investigations of episodic memory have reported an overall positive effect of mindfulness, but mixed findings on the process responsible for this outcome. Indeed, Brown et al. (2016) observed an enhancement of memory performance in recognition and free-recall tasks after 10 min of meditation practice before the encoding and the testing phases compared to participants listening white-noise or waiting without any specific instructions. On the other hand, Lloyd et al. (2016) reported lower false-recognition rates following 3 min of meditation practice than after listening to a story. However, this positive effect of mindfulness was observed only when mindfulness practice occurred before recognition but not before encoding.

Concerning dispositional mindfulness, Rosenstreich and Ruderman (2016) found a negative correlation between response bias and the facet “Non-judging” (e.g., adopting a neutral attitude toward one’s thoughts and actions), assessed with a short form of the Five Facet Mindfulness Questionnaire (FFMQ; Baer et al., 2006). These results suggest that dispositional mindfulness would modulate decision criterion rather than encoding processes. Thus, while the overall effect of mindfulness meditation on memory seems to be positive, the precise mechanism leading to enhanced memory performances is still unclear. Some studies suggest an improvement of encoding (Brown et al., 2016; Lykins et al., 2012), and others of retrieval processes (Hargus et al., 2010; Lloyd et al., 2016; Rosenstreich & Ruderman, 2016; Williams et al., 2000).

### False memories

The picture is made even less clear by more recent findings showing increased false-memory susceptibility following mindfulness meditation. Interestingly, false-memory production is not necessarily related to memory impairements but could reflect a healthy and normal functioning of memory processes as illustrated by Budson et al. (2000) who observed a lower level of false memories production in Alzheimer patients.

Different types of false memories exist and have been the topic of flourishing literature (Langevin et al., 2009). To our knowledge, the investigations concerning the effect of mindfulness on false memories have only used the well-known Deese–Roediger–McDermott (DRM) paradigm (Deese, 1959; Roediger & McDermott, 1995), probably the most employed experimental protocol to induce false memory in a laboratory setting. In this experimental manipulation, participants study lists of words semantically related, where the most "stereotypical" item of each list, the "critical item", is voluntarily omitted. The critical item can be either the most representative item for categorical lists (e.g., car for vehicles) or characterize by its semantic associative strength with other items (e.g., cold for winter, ice, snow, freeze, frost). This procedure usually generates a high rate (about 50%) of false recall or recognition of the critical items (Langevin et al., 2009).

According to the Activation Monitoring Theory (AMT; Gallo & Roediger, 2002), two mechanisms could be responsible for producing false memories. First, during the encoding process, the study of semantically related words would activate the critical item due to its association with the studied material. Second, during the retrieval phase, a failure of monitoring processes would prevent the correct rejection of the critical lure (Gallo, 2010). The Fuzzy-Trace Theory (FTT) suggests another explanation (Brainerd & Reyna, 2002). According to the FTT, two types of memory traces could coexist: the gist, build from the conceptual and semantic information, and the verbatim, related to specific details (perceptual and contextual). Consequently, false memories would result from the reliance on shared gist information between critical and studied items and the failure to discriminate between studied and unstudied items due to the inability to retrieve the verbatim, considered more labile.

In line with the FTT predictions, the increase of salient perceptual information or item distinctiveness can reduce false memories in the context of DRM due to their strengthening of the verbatim trace (for a review see, Huff et al., 2015). Item distinctiveness can be manipulated, for example, by varying the font of each studied word (e.g., Arndt, 2006) or using pictorial material (e.g., Abichou et al., 2020; Foley & Foy, 2008; Israel & Schacter, 1997; Olszewska & Ulatowska, 2019; Abichou et al., 2021): when verbatim information is available, false memories decrease. Although it is not always the case, as suggested by the observations made by Drowos et al. (2010), with patients presenting posterior parietal cortex (PPC) damages. Interestingly, while the patients presented a lower rate of false recognition compared to a control group for auditory material, the addition of perceptual information did not improve their performances, leading to a higher rate of false recognition compared to healthy individuals. According to Drowos et al. (2010), these results could reflect an incapacity to use verbatim information during retrieval processes rather than a failure to encode this information.

### Mindfulness and false memories

Wilson et al. (2015) conducted one of the first studies investigating the impact of mindfulness meditation on false memory. Using 15 min of meditation practice compared to mind-wandering instructions before the encoding, they observed an increase of false recall and false recognition after meditation practice. Additionally, in this study, mindfulness fails to improve correct free-recall and recognition contrary to previous observations (Brown et al., 2016). To explain such results, Wilson et al. (2015) proposed that mindfulness induced a failure of the cognitive control during the retrieval processes due to the adoption of a more liberal response bias, leading to consider erroneously critical items as studied.

Similarly, Rosenstreich and Ruderman (2017) suggested that mindfulness could increase retrieval based on semantic processes or familiarity rather than perceptual sources or recollection processes, which would impair monitoring processes in the specific case of DRM. Sustaining this hypothesis, Rosenstreich (2016) reported two studies observing an increase of false recognition following one to five sessions of 30 min of mindfulness practice, compared to a waitlist or a mind-wandering control condition. Nevertheless, and contrary to Wilson et al. (2015), Rosenstreich (2016) also reported an increase in correct recognition following mindfulness practice. The allocation of attentional resources during the encoding phase could explain these seemingly counterintuitive results. Indeed, reducing attentional resources during encoding could diminish the rate of false recognition by preventing the critical item’s activation (Knott & Dewhurst, 2007). Therefore, by enhancing the availability of attentional resources, mindfulness meditation facilitates semantic activation, thus increasing the rate of false memories and correct recognition.

However, recent studies failed to replicate these findings. Comparing the effect of 15 min of mindfulness practice to either mind-wandering instructions or an alternative control group (word search puzzles), Baranski and Was (2017) did not observe any effect on correct or false recognition related to the mindfulness condition. Nevertheless, these experimental manipulations also included a “warning conditions”, where participants were alerted of the potential presence of lures, suggesting a more substantial effect of this alert than the mindfulness induction.

On the other hand, comparing mindfulness induction to a control group not receiving meditation instructions, Calvillo et al. (2018) observed a positive effect of meditation on response bias (e.g., more conservative) when mindfulness induction took place after the encoding. Similarly to Baranski and Was (2017), Sherman and Grange (2020) did not observe any effect either on correct or false recognitions of a 15-min mindfulness meditation practice compared to a mind-wandering induction or a join-the-dots task taking place before the encoding. To explain these inconsistencies, Sherman and Grange (2020) suggest that mind-wandering might not be an optimal control condition, despite its widespread use in studies investigating the impact of a single mindfulness meditation session. They observed that participants from mindfulness and control conditions reported similar mental states between the two inductions on the State Mindfulness Scale (Tanay & Bernstein, 2013) and the Retrospective Mind-wandering Scale from the Dundee Stress State Questionnaire (Matthews et al., 1999). These results are consistent with recent critics of mindfulness research, arguing that mind-wandering could induce a similar cognitive state as mindfulness in participants naïve to meditation practice (Davidson & Kaszniak, 2015).

To our knowledge, only one study investigated the impact of dispositional mindfulness on false memory in the DRM context (Yeh & Lu, 2017). In this study, a set of Chinese and English words were used in the encoding and the retrieval phase, either in congruent (e.g., English at encoding and retrieval) or incongruent (e.g., Chinese at encoding and English at retrieval) conditions. While the authors observed a positive correlation between dispositional mindfulness and memory sensitivity in the congruent condition, they reported an opposite pattern when the languages at encoding and recognition were different. According to Yeh and Lu (2017), dispositional mindfulness could impact false memories formation by two different processes. When both verbatim and gist information is available, such as in the congruent condition, mindfulness will decrease false-recognition rates by enhancing verbatim information availability. On the contrary, in the incongruent condition, when only gist information is available, participants with high dispositional mindfulness, via an increased activation in the semantic network, would rely on a more strongly activated gist trace and incorrectly accept critical items. Altogether, these results suggest that the impact of mindfulness on false memories would depend on the distinctiveness of the studied material. This assumption is in line with the systematic analysis conducted by Konjedi and Maleeh (2020), suggesting a positive correlation between dispositional mindfulness and perceptual acuity, enhancing the quality and accuracy of encoded information. Thus, by promoting the encoding of perceptual features, mindfulness could improve source-monitoring and memory sensitivity.

### Current investigation

The present study aims to fulfil several goals. First, we wanted to replicate and generalize the effect of mindfulness on producing false memories to visual material. As suggested by previous studies, the nature of the encoded material must be considered when assessing the impact of mindfulness on false-memory generation. According to the aforementioned studies, our main hypothesis was that employing visual material, contrary to verbal material, should decrease false-memory rate, particularly after a short induction of meditation, as suggested by Konjedi and Maleeh (2020). In that respect, if mindfulness elicits encoding processes based on external processing or verbatim, the presence of perceptual cues should improve the ability to discriminate studied items from critical items. Second, previous studies investigating the link between mindfulness and false memory have not systematically considered the role of dispositional mindfulness. Only the study from Yeh and Lu (2017) questioned this link in the DRM context. Nevertheless, they employed the Mindful Attention Awareness Scale (MAAS; Brown, & Ryan, 2003), measuring only one aspect of dispositional mindfulness, namely, ones’ moment-to-moment receptive attention in daily life. However, more recent conceptualizations consider mindfulness as a multi-dimensional construct (Baer et al., 2006). In the context of studied items possessing higher distinctiveness, we expect to observe a positive correlation between dispositional mindfulness and memory sensitivity, but a negative correlation with response bias, as suggested by Yeh and Lu (2017) observations.

To test these hypotheses, we employed the FalseMem task (Abichou et al., 2020; Abichou et al., 2021), a virtual reality adaptation of the DRM paradigm. Immersed in a virtual environment, participants had to walk among 3D rendered DRM lists materialized as retail shops. Participants were explicitly asked to pay attention to the presented items and voluntary memorize them. Before the encoding in virtual reality, participants listened to either a short mindfulness induction or a control condition (listening to a story). We selected this control condition following the recent critics targeting the problematic uses of mind-wandering as a control condition for mindfulness researches (Girardeau et al., 2020; Sherman & Grange, 2020). This alternative control condition has already been successfully used in several studies (Kramer et al., 2013; Lloyd et al., 2016; Zeidan et al., 2010). Finally, we used the Five Facets Mindfulness Questionnaire (Baer et al., 2006) to assess dispositional mindfulness. To our knowledge, this is the first study investigating the impact of mindfulness on false-memory production for complex visual material and memory performances in conditions closer to real life.

## Materials and methods

### Participants

Participants were recruited at the Institute of Psychology at the University of Paris Descartes and received credits for their participation. To be eligible, participants should: (1) be aged between 18 and 30 years; (2) without any neurological or psychiatric history; (3) without any auditory or visual troubles preventing the use of virtual reality; (4) without any substances addictions; (5) not practicing meditation, and (6) be native French speakers. We recruited 44 participants, randomly assigned to mindfulness (*N* = 22, 16 females, age 21.20 ± 2.26 years) or a control (*N* = 22, 19 females, age 21.88 ± 3.15 years) condition (listening to a story). Participants in the two groups were matched on several cognitive and emotional variables (see Table 1). We also verified that participants in the two groups did not differ in their level of dispositional mindfulness (see Table 2).

**Table 1:** Descriptive statistics and between groups comparison of the cognitive and emotional assessments

	Mindfulness	Story	*p* value
Age	21.20 (2.26)	21.88 (3.15)	0.41
Mood—arousal	5.77 (1.45)	5.32 (1.96)	0.39
Mood—valence	6.23 (2.14)	6.32 (1.64)	0.88
BDI	5.27 (5.12)	6.41 (4.77)	0.45
Trail Making Test (TMT)	0.36 (0.98)	0.21 (0.73)	0.57
Digit span	17.13 (3.04)	16.91 (3.61)	0.82

Between parentheses is reported the standard deviation

*BDI* beck depression inventory

**Table 2:** Descriptive statistics and between groups comparison of the FFMQ scores

	Mindfulness	Story	*p* value
Acting with Awareness	3.28 (0.69)	3.18 (0.69)	0.65
Describing	3.47 (0.64)	3.16 (0.75)	0.14
Observing	3.41 (0.58)	3.14 (0.77)	0.20
Non-judging	2.97 (0.89)	2.95 (0.73)	0.93
Non-Reactivity	2.98 (0.66)	3.02 (0.76)	0.86

Between parentheses is reported the standard deviation

The study used a double-blind protocol. We recruited participants through an advertisement about the effect of relaxation on cognitive performances. The experimenters were blind about the condition assigned to each participant. The protocol was carried out under the local ethical standards. Participants were informed of the academic nature of the study and accepted that their data would be processed anonymously. After a full explanation of the procedure, all participants gave written informed consent before carrying out the study.

### Questionnaires and neuropsychological assessment

To ensure the matching between our two experimental groups, we assessed participants’ mood and cognitive functions.

#### Questionnaires

To assess dispositional mindfulness, we used the Five Facet Mindfulness Questionnaire (FFMQ; Baer et al., 2006; French adaptation Heeren et al., 2011). Composed of 39 items (5-point Likert scales), the FFMQ evaluates five sub-components associated with mindfulness: (1) Observing (tendency to pay attention to feelings and surroundings), (2) Describing (ability to describe feelings), (3) Acting with Awareness (degree of attention toward what one does), (4) Non-judging (adopt a neutral attitude toward one’s thoughts and actions), (5) Non-Reactivity (tendency to not react to thoughts and feelings). Besides, the level of depression, known to affect memory performances, was assessed by the short version of the Beck Depression Inventory (BDI; Collet & Cottraux, 1986). Finally, the participants’ mood arousal and valence were assessed before and after the meditation induction and the control conditions using a matrix inspired by Colzato et al. (2012).

#### Neuropsychological assessment

The Trail Making Test part A and B (TMT; Lezak et al., 2004) was employed to assess cognitive flexibility. The digit span, backward and forward, from the MEM-III (Wechsler, 2001) was employed to assess working memory capacity and attentional resources.

### Inductions

We created two audio-tracks lasting 15 min for our experimental conditions, recorded by the same male voice. Both audio-tracks are available online (https://bit.ly/2Jinsav and https://bit.ly/2kxVmtU). During the mindfulness session, participants were instructed to focus on their breathing and notice when their mind wandered from the chosen object of attention. At this moment, they were encouraged to redirect their attention to their breathing. The same mindfulness induction was already employed by Girardeau et al. (2020). The control condition consisted of a philosophical tale where participants were instructed to listen and pay attention to the narrative. The randomization was performed automatically by the Neuropsydia software (Makowski & Dutriaux, 2017).

### Manipulation checks

After listening to the audio-tracks, a series of six visual analogical scales (ranging from 0 to 100) was completed by participants to evaluate their cognitive state during the induction. Participants were asked to report their level of sleepiness (“I felt sleepy”), mind-wandering (“My thoughts freely wander, without control”), focused attention (“I was focused on a specific idea, sensation or perception), internal absorption (“My attention was caught by events that I imagined or remembered”), body-awareness [“My attention was focused on my body sensations (e.g., breathe, heartbeat)”], and external absorption [“My attention was caught by what was happening around me (e.g., sounds, voices)”]. The items presented in this study were the same as those used by Girardeau et al. (2020).

### Memory task in virtual reality: FalseMem Task

For the memory test, we employed the FalseMem task (Abichou et al., 2020; Abichou et al., 2021), which allows a naturalistic assessment of true and false memory both in free recall and recognition. The encoding phase of the FalseMem task was implemented in a 3-D realistic environment build with the Unity 3D software in the Memory, Brain and Cognition lab. A sample of the environment is available online: https://osf.io/4znwb/?view_only=34476f5c6003467986763a9cb8e03881.

Participants navigated, using a gamepad, through a virtual city presented on a computer laptop (Asus, Republic of Gamers, 17 inches screen). Before starting the experimental session, participants completed a series of tasks in a virtual training space to ensure their ability to navigate a 3-D environment using the gamepad. For the encoding phase, we provided precise instructions indicating to participants to pay attention to the stalls identified by a red signal on the floor. Besides, we warned participants that a memory test would proceed afterwards. The stalls corresponded to seven categorial DRM lists: animals, fruits, vegetables, musical instruments, furniture, clothes, and tools. Each list was composed of ten items, except for the category “animals” that, for technical reasons, comprised only nine items. The time required to complete the journey across the different stalls was recorded for each participant to control the exposure to the environment. After the encoding phase, we conducted a free recall of the items composing the DRM lists. During this session, participants had 10 min to recall as many items as presented in the stalls. Immediately after the free recall, participants performed the FalseMem recognition task where 157 items were presented using Neuropsydia software (Makowski & Dutriaux, 2017): 69 targets items presented in the virtual environment; 7 critical items (one for each DRM list), corresponding to the most representative item of the categorical list; 28 semantically related items; 28 perceptually associated items; and 25 neutral items. Items were randomly presented, self-paced by participants and delivered on a computer screen with a black background. For each item, participants had to determine if it was presented or not in the virtual environment. When participants considered an item as previously studied, they were required to precise the nature of the state of consciousness associated with the recognition using the R/K/G paradigm (Gardiner et al., 1998).

Before the recognition task, we instructed participants to respond, “I remember” (R), only for a vivid reminiscence of items associated with contextual information. The answer “I know” (K) was associated with a feeling of familiarity, without a recollection of perceptual information. Finally, we instructed participants to answer, “I guess” (G) when they were not sure about their answer, reflecting a lower degree of certainty related to inferential rather than memory processes.

### Procedure

We conducted each session individually. After explaining the experimental procedure, we asked participants to sign an agreement to participate in the study. The protocol started with the self-report questionnaires and the neuropsychological assessments in the following order: the matrix to collect their current mood, the FFMQ, the BDI, the TMT and the digit span. After this first phase, participants discovered the virtual training environment and executed several timed tasks to familiarize themselves with the gamepad. Following this, we introduced the instructions for the encoding task. After that, the induction phase started with a short anamnesis, including questions about age, sex, education level, and check if our participants had any previous meditation practice. After, participants were randomly assigned by the software to the mindfulness or the story induction. Participants were equipped with an audio headset and left alone, in front of the computer screen during the induction. At the end of the audio-track, participants completed the manipulation check items that were automatically displayed. Then, we used the same matrix as the beginning of the experimentation to assess participants’ mood to test potential changes after the induction. After a short reminder of the navigation instructions, participants started their walk in the virtual environment corresponding to the encoding phase. Immediately after, we proceed to the free-recall task and the recognition task. Finally, we conducted a short debriefing to provide details and information about the procedure to our participants. The overall duration of the experimental protocol was about 1 h and 30 min.

## Data analyses

We checked the effect of our experimental manipulations in several ways. First, we run two separate 2 × 2 mixed ANOVA with the time of measure (before and after the induction) and induction (mindfulness and story) as factors on participants’ mood valence and arousal. Second, we compared the score on each question of the manipulation check questionnaire administered immediately after the induction between our two experimental conditions with independent sample *t*-tests (two-tailed). Then, we also checked the impact of the induction on the navigation time. Since we found a significant difference, with *t*(42) = 2.16, *p* < 0.05, 95% CI [5.11; 152.44] between the two groups, with participants in the mindfulness group having a longer navigation time (578.59 ± 135.41 s) than those in the control group (499.82 ± 104.23 s), we added this variable as a covariate in all subsequent analyses.

For the free recall, we calculate the correct recall rate and false recall rate for each participant by dividing the number of correct recall items by the total number of items presented (69) and by dividing the number of false recalls by the total number of critical items (7), respectively.

For the recognition, we computed several scores: (1) the hit rate, obtained by dividing the total number of correct recognitions by the total number of target items (69); (2) a false-recognition rate per items category, calculated by dividing the number of false recognitions by the number of lures presented during the recognition task. Thus, the number of false recognitions was divided by 25 for neutral, 28 for perceptual and semantic, and 7 for critical items.

For the Signal Detection Theory (SDT) indexes, we used the non-parametric indexes of response bias (bppd) and discrimination (A′), which have a lower sensitivity to extreme values and do not make assumptions on the signal or noise distributions (Pallier, 2002). We applied Pallier’s (2002) formula to compute global A′ and bppd scores and calculated these scores for each type of items per participant. We computed A′ and bddp scores based on hit rate and false-recognition rate per items category, likewise for false-recognition rates (see previous paragraph). In memory process studies, the A′ score translated the capacity to discriminate old from new items. A′ scores close to 0.5 indicate chance level of discrimination, while higher scores correspond to better discrimination abilities. Bppd scores indicated the tendency of the participants to adopt a liberal or conservative decision criterion. A positive score reveals a conservative decision criterion (tendency to answer no), and a negative score is related to a liberal decision criterion (tendency to answer yes).

Finally, using the formulas suggested by Rosentreich and Ruderman (2017), we transformed the R/K/G responses in a recollection and a familiarity score to capture our participants' mental operations during the recognition phase (Yonelinas & Jacoby, 2012). A summary of the descriptive statistics of all memory scores is provided in Table 3.

**Table 3:** Descriptive statistics of the memory measures

	Mindfulness	Story	*p* value
Correct recall	0.32 (0.14)	0.28 (0.11)	0.86
False recall	0.09 (0.12)	0.09 (0.10)	0.84
Hit recognition	0.54 (0.16)	0.51 (0.14)	0.56
False recognition	0.16 (0.08)	0.19 (0.09)	0.24
A′	0.76 (0.11)	0.73 (0.13)	0.02*
Bddp	0.54 (0.43)	0.53 (0.44)	0.95
Recollection hit	0.40 (0.14)	0.33 (0.14)	0.07
Recollection false	0.07 (0.09)	0.05 (0.08)	0.30
Familiarity hit	0.17 (0.13)	0.18 (0.13)	0.87
Familiarity false	0.06 (0.09)	0.07 (0.11)	0.97

Between parentheses is reported the standard deviation

We ran an ANCOVA analysis for the hit rate, with the induction (mindfulness and story) as a between-subject factor. For the false-recognition rate, the SDT indexes (A′, bppd), and the familiarity and recollection scores, we used a mixed ANCOVA with the induction (mindfulness and story) as a between-subject factor and the type of lure (neutral, semantic, perceptual, and critical) as a within-subject factor. For all post hoc comparisons, we used the Hölm correction for multiple comparisons.

Finally, we conducted separate stepwise multiple regressions to predict each of our dependent variables with the 5 FFMQ subscales. All statistical analyses were conducted using R software (3.4.4 version). The R scripts used for data analyses and the complete dataset are available on the Open Science Framework repository (https://osf.io/2yj9n/).

## Results

### Manipulation checks

First, concerning participants’ mood, we did not observe any effect of the induction for the valence with *F*(1,84) = 0.00, *p* = 1.00, *η*^2^ < 0.01; neither a main effect of time, with *F*(1,84) = 1.22, *p* = 0.27, *η*^2^ = 0.01. Moreover, the interaction between those factors was not significant with *F*(1,84) = 0.05, *p* = 0.83, *η*^2^ < 0.01.

For the arousal, we observed a main effect of time, *F*(1,84) = 13.98, *p* < 0.01, *η*^2^ = 0.14, overall participants reported lower level of arousal after the two inductions (4.05 ± 2.06), compared to their level of arousal before (5.55 ± 1.72). The main effect of the induction was not significant with *F*(1,84) = 0.32, *p* = 0.57, *η*^2^ < 0.01. The interaction between the two factors was not significant, with *F*(1,84) = 2.89, *p* = 0.09, *η*^2^ = 0.03.

Second, we checked the impact of the inductions on the cognitive state of our participants. After the mindfulness induction, participants displayed higher score on body-awareness (63.26 ± 22.75), with *t*(42) = 5.89, *p* < 0.001, 95% CI [27.00; 55.15] than after the story induction (22.18 ± 25.50). Additionally, participants who had followed the mindfulness induction reported higher scores on the focused attention question (51.32 ± 28.41), with *t*(42) = 3.60, *p* < 0.01, 95% CI [12.25; 43.59] than participants listening the story audio-track (23.40 ± 22.71). At last, the level of sleepiness was higher for the mindfulness induction (64.10 ± 21.71), with *t*(42) = 3.30, *p* < 0.01, 95% CI [9.15; 37.92] than for the story induction (40.56 ± 25.39). A lack of significant differences between the two conditions was observed for the internal absorption with *t*(42) = 0.89, *p* = 0.38, 95% CI [1.00; 25.69] and for mind-wandering with *t*(42) = 1.10, *p* = 0.28, 95% CI [− 8.72; 29.70]. For full descriptive statistics, see Table 4 in Supplementary Material.

### Free-recall

No significant difference was found between the mindfulness induction (0.32 ± 0.14) and *story* (0.28 ± 0.11) conditions for the ratio of correct recalled item with *F*(1,41) = 0.03, *p* = 0.86, *η*^2^ < 0.01, neither for the ratio of false recall with *F*(1,41) = 0.04, *p* = 0.84, *η*^2^ < 0.01; mindfulness induction (0.09 ± 0.12) and story (0.09 ± 0.10).

### Hit and false-recognition rates

**Fig. 1:**
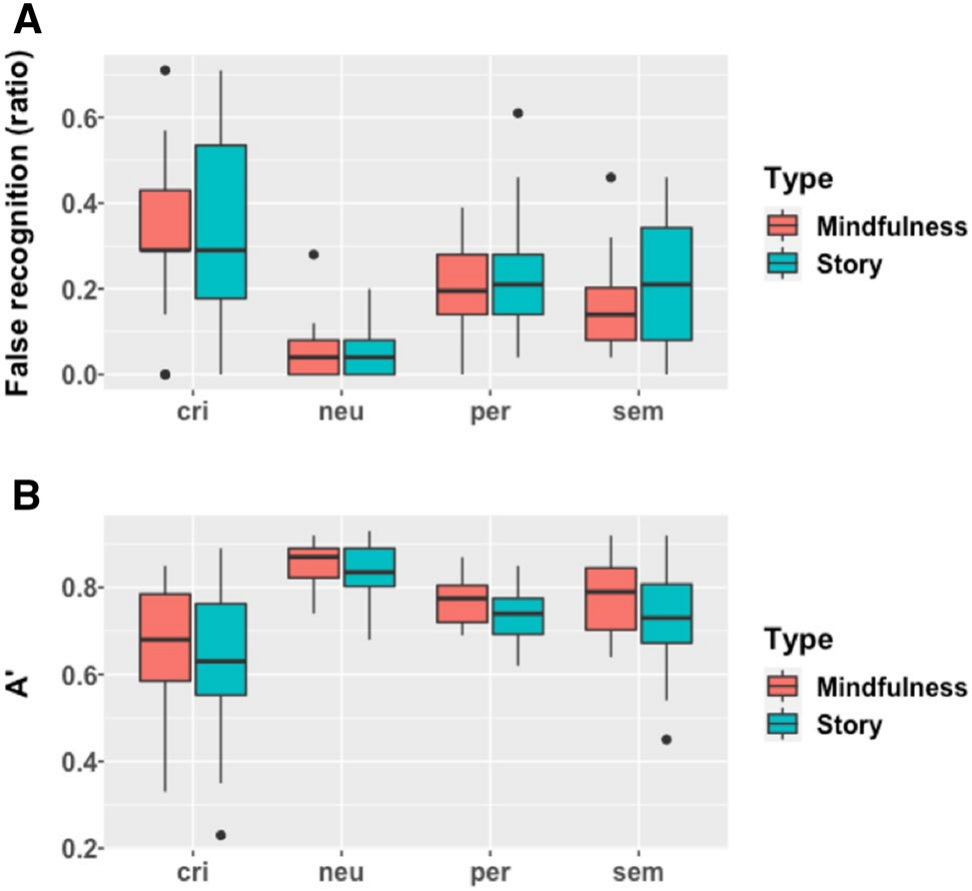
Box plot of memory performances by types of lures and type of induction. **a** False recognition rate. **b** Discrimination index (A′)

For the correct recognition rate (hits), no significant difference was reported between mindfulness induction (0.54 ± 0.16) and story (0.51 ± 0.14) with *F*(1,41) = 0.34, *p* = 0.56, η^2^ < 0.01. For the false-recognition rate, we found a significant main effect of the type of items on false recognition with *F*(3,167) = 35.51, *p* < 0.001, η^2^ = 0.37. The post hoc analyses revealed that false-recognition rate of critical items (0.34 ± 0.20) was significantly higher than semantic (0.18 ± 0.13, *p* < 0.001), perceptual (0.21 ± 0.12, *p* < 0.001) and neutral items (0.06 ± 0.06, *p* < 0.001). Moreover, false-recognition rates of neutral items were significantly lower (all *p* < 0.001) than perceptual and semantic items (see Fig. 1a and Supplementary material). The difference between perceptual and semantic items was not significant (*p* = 0.30). The main effect of the induction was not significant with mindfulness induction (0.19 ± 0.16) and story (0.21 ± 0.18) with *F*(1,167) = 1.79, *p* = 0.18, *η*^2^ < 0.01. At last, the interaction between the induction and the type of items was not significant with *F*(3,167) = 0.29 *p* = 0.83, *η*^2^ < 0.01.

### SDT indexes

For the discrimination index (A′), a main effect of the induction was observed with *F*(1,167) = 5.88, *p* = 0.02, η^2^ < 0.01, indicating a significant difference between mindfulness induction (0.77 ± 0.11) and story (0.73 ± 0.13). The main effect of the type of items was also significant with *F*(3,167) = 31.73, *p* < 0.001, η^2^ = 0.33. The post hoc analyses revealed that discrimination index was significantly lower for critical items (0.65 ± 0.15) compared to semantic (0.76 ± 0.11, *p* < 0.001), perceptual (0.75 ± 0.06, *p* < 0.001), and neutral items (0.85 ± 0.06, *p* < 0.001). Moreover, discrimination index was significantly higher for neutral items, compared to perceptual and semantic items (all *p* < 0.001). The difference between perceptual and semantic items was not significant (*p* = 0.80). See Fig. 1b and see Supplementary material. A lack of significant interaction between the induction and the type of items was also reported with *F*(3,167) = 0.29, *p* = 0.83, *η*^2^ < 0.01.

For the response bias, we found a significant main effect of the type of items with *F*(3,167) = 20.50, *p* < 0.001, *η*^2^ = 0.26. The post hoc analyses revealed a significant lower score for critical items (0.24 ± 0.50) compared to semantic (0.58 ± 0.37, *p* < 0.001), perceptual (0.47 ± 0.39, *p* = 0.01) and neutral items (0.85 ± 0.18, *p* < 0.001). Moreover, the score was higher for neutral items, compared to perceptual and semantic items (all *p* < 0.001). The difference between perceptual and semantic items was not significant (*p* = 0.18), see Supplementary material. We did not find any significant effect of the induction with *F*(1,167) < 0.01, *p* = 0.95, *η*^2^ < 0.01, and no significant interaction with *F*(3,167) = 0.20, *p* = 0.90, *η*^2^ < 0.01.

### Recollection and familiarity

For the correct recognitions, we found a trend effect of the induction on the recollection scores with *F*(1; 41) = 3.54, *p* = 0.07, *η*^2^ = 0 0.03, with mindfulness induction (0.40 ± 0.14) having a higher score than story (0.33 ± 0.14). For the familiarity scores, we did not find any effect of the induction with *F*(1; 41) = 0.03, *p* = 0.87, *η*^2^ < 0.01.

For the false recognition, we found a significant main effect of the type of items on the recollection scores, with *F*(3; 167) = 13.50, *p* < 0.001, *η*^2^ = 0.19. The post hoc analyses revealed a greater recollection score for critical items (0.10 ± 0.14), compared to semantic (0.04 ± 0.05, *p* < 0.01), perceptual (0.09 ± 0.05, *p* = 0.01), and neutral items (0.01 ± 0.02, *p* < 0.001). Moreover, neutral items were associated with a lower recollection score compared to perceptual items (*p* < 0.001) and semantic items (*p* = 0.05). The difference between perceptual and semantic items was not significant (*p* = 0.18), see Supplementary material. The main effect of the induction with *F*(1; 167) = 1.08, *p* = 0.30, *η*^2^ < 0.01, and the interaction with *F*(3; 167) = 1.06, *p* = 0.37, *η*^2^ < 0.05 were not significant.

For the familiarity scores, we found a main effect of type of items, with *F*(3; 167) = 9.67, *p* < 0.001, η^2^ = 0.14. The post hoc analyses revealed greater scores for critical (0.16 ± 0.12) compared to semantic (0.06 ± 0.07, *p* = 0.02), perceptual (0.06 ± 0.06, *p* = 0.05), and neutral items (0.01 ± 0.03, *p* < 0.001). Scores of familiarity were also significantly lower for neutral compared to perceptual (*p* = 0.05) and semantic items (*p* = 0.05) items. The difference between perceptual and semantic items was not significant (*p* = 1.00), see Supplementary material. The main effect of the induction with *F*(1; 167) < 0.01, *p* = 0.97, η^2^ < 0.01 and the interaction with *F*(3; 167) = 0.16, *p* = 0.92, *η*^2^ < 0.01 were not significant.

### Dispositional mindfulness

**Fig. 2:**
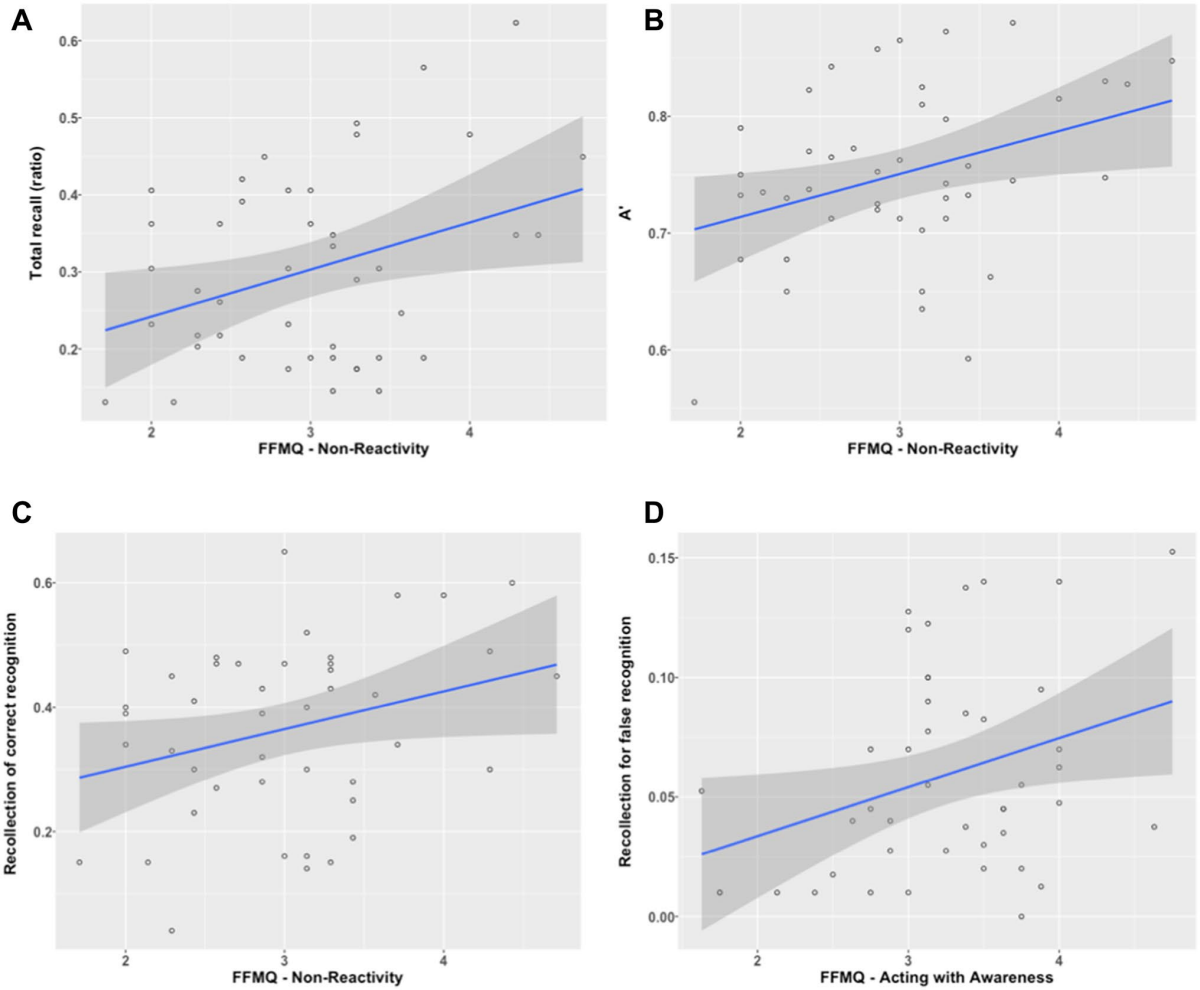
Regression lines between FFMQ facets and memory performances. **a** Relationship between the Non-Reactivity facet of mindfulness and total recall rate. **b** Relationship between the Non-Reactivity facet of mindfulness and A′ index. **c** Relationship between the Non-Reactivity facet of mindfulness and recollection of correct recognition. **d** Relationship between the Acting with Awareness facet of mindfulness and recollection of false recognition. Solid lines represent regression lines and transparent grey areas represent 95% confidence interval

For the free recall, the facet Non-Reactivity significantly predicted correct recall rate with *b* = 0.06, *p* = 0.02, *η*^2^ = 0.12. The global model was significant with *F*(1; 42) = 5.78, *p* = 0.02, adjusted *R*^2^ = 0.10 (see Fig. 2). None of the facets significantly predicted the false recall rate, with the global model being non-significant with *F*(1; 42) = 2.99, *p* = 0.09, adjusted *R*^2^ = 0.04.

For the correct recognition rate, we did not observe any effect of the facets, with *F*(1; 42) = 2.88, *p* = 0.10, adjusted *R*^2^ = 0.04. The same results were reported for the false-recognition rate, with *F*(3; 40) = 2.00, *p* = 0.13, adjusted *R*^2^ = 0.07.

For the SDT indexes, the facet Non-Reactivity positively predicted to the A′, with *b* = 0.04, *p* = 0.02, *η*^2^ = 0.14, with the global model being significant with *F*(1; 42) = 5.86, *p* = 0.02, adjusted *R*^2^ = 0.10 (see Fig. 2). None of the facets significantly predicted the response bias with *F*(2; 41) = 1.74, *p* = 0.19, adjusted *R*^2^ = 0.03.

For the recollection score, a positive effect was observed for the facet Non-Reactivity for the correct recognition, with *b* = 0.06, *p* = 0.05, *η*^2^ = 0.08, with the global model being significant and *F*(2; 41) = 4.11, *p* = 0.05, adjusted *R*^2^ = 0.07 (see Fig. 2). We also found that the facet Acting with Awareness positively predicted the recollection score for the false recognition with *b* = 0.02, *p* = 0.03, *η*^2^ < 0.01, with the global model being significant *F*(1; 42) = 5.07, *p* = 0.03 and adjusted *R*^2^ = 0.09 (see Fig. 2). For the familiarity score, none of the facets significantly predicted the familiarity score for the correct recognitions, with *F*(1; 42) = 2.5, *p* = 0.12 and adjusted *R*^2^ = 0.03, neither for the false recognitions, with *F*(5; 38) = 0.93, *p* = 0.47 and adjusted *R*^2^ = − 0.01.

## Discussion

False memories have been extensively studied either in false recall and false-recognition tasks to investigate the memory process occurring during encoding and retrieval phases, highlighting healthy and pathological functions. The present study aimed to investigate the impact of state and dispositional mindfulness on false memories production using an adaptation of the classical DRM task into virtual reality. Our main results were that mindfulness induction did not affect the rate of false recall or false recognition. Nevertheless, the mindfulness induction significantly improved memory sensitivity as witnessed by higher discrimination, measured with the A′, than following the control induction. Moreover, we found a positive and negative effect of dispositional mindfulness on memory performances. Indeed, the facet Non-Reactivity positively predicted the total recall of items, the recollection score of studied items and the discrimination index. Overall, we found a positive association between state and dispositional mindfulness with memory capacities by improving recall, recollection and memory sensitivity. Although, a potential side-effect of the facet Acting with Awareness was also observed, with a positive correlation with the recollection score of items incorrectly recognized as old.

Our null findings on the impact of mindfulness meditation on false memories productions is in contradiction with previous studies reporting either an increased susceptibility (Rosentreich, 2016; Rosentreich & Ruderman, 2017; Wilson et al., 2015) or a reduction in false recalls or recognitions (Baranski & Was, 2017; Calvillo et al., 2018).

A failure of our experimental manipulation cannot easily explain these null findings. Indeed, participants reported being more aware of their body sensations and having a more stable attentional focus after a short meditation induction than listening to a short story. Thus, the cognitive state reported by our participants after the mindfulness induction was in the expected direction. Moreover, participants in the mindfulness induction reported being sleepier. Consistent with the proposal that participants naïve to meditation can find the exercise more effortful and thus feel more fatigue and sleep propensity (Britton et al., 2014), previous studies have already reported increased sleepiness after a brief mindfulness induction (Lin et al., 2020). Besides, our results are in line with other studies failing to report any significant effect of a short mindfulness induction on false memories (Baranski & Was, 2017; Sherman & Grange, 2020). Nevertheless, a direct comparison with the aforementioned studies is not straightforward due to methodological differences. In that respect, Wilson et al. (2015) and Rosentreich (2016) have used the ‘classical’ DRM paradigm, with a mindfulness induction before the encoding compared to a mind-wandering control condition. Baranski and Was (2017) used a similar procedure but, in one experiment, warned participants about the presence of potential lures, while Calvillo et al. (2018) delivered the induction after the encoding. More recently, Sherman and Grange (2020) used puzzle-solving instead of mind-wandering as a control condition. One likely explanation for these heterogeneous results is that the effect of mindfulness meditation on false memory is not robust and generalizable to all conditions. As a matter of facts, the present study employed pictorial instead of verbal material. As mentioned in the introduction, such material usually improves memory performances due to the availability of perceptual information, allowing the discrimination of studied item from lures. In that respect, our translation from a classical DRM material to a 3-D realistic environment could explain the lack of reproducibility of previous studies due to the addition of perceptual cues. The material used in this study could also explain why we reported that participants in the mindfulness meditation condition had higher memory sensitivity than the control group. This latter finding echoes those reported on the effect of brief mindfulness meditation on episodic memory (Brown et al., 2016) and extend it to visual material encoded in a complex environment. We also reported a trend toward a higher score of recollection for correct recognition after the mindfulness induction. This finding is again in line with Brown et al. (2016) reporting that mindfulness was linked only to higher memory sensitivity for ‘remember’, but not for ‘know’, responses. Moreover, contrary to Rosentreich and Ruderman (2017), we did not find a significant effect of mindfulness meditation on response bias. Altogether, our results seem to contradict Levi and Rosentreich (2019) recent proposition that mindfulness would affect memory performances mostly by changes in decision-making processes.

Nevertheless, we did not observe a significant effect of mindfulness on correct recall or recognition despite better memory accuracy scores. Thereupon, the DRM translation into a virtual environment generated an overall decrease in memory performances compared to a classical verbal material with around 30% of correct recall and 50% correct recognition. In that respect, the media richness of virtual reality could have led to a higher cognitive load in perceptual information to encode, since DRM lists and other irrelevant items composed our environment (e.g., virtual avatars, road traffic, buildings). According to Schrader and Bastiaens (2012), virtual environments could lower memory performances partly because of the cognitive load induced by the sense of presence, characterized by feeling immersed in the 3-D environment and react as if it was real. Nevertheless, more than being a limitation, this seems to mirror memory performances for real-life events. Indeed, Misra et al. (2018) observed that participants did not retain much of the details of their everyday life experiences. These findings stress the limitations of classical investigation of memory processes using laboratory material (e.g., words or pictures lists). In line with this assumption, La Corte et al. (2019) pointed out that virtual reality could provide a greater ecological validity to investigate memory processes and tackle these current methodological limitations.

Concerning dispositional mindfulness, we found that different facets could lead to opposite effects on memory performances. On the one hand, the facet Non-Reactivity positively predicted the correct recall ratio, the recollection score of studied items and the discrimination index (A′). Thus, this facet globally predicted better memory performances. On the other hand, the facet Acting with Awareness increased the recollection score associated with lures false recognition. Some studies suggest that Non-Reactivity is associated with cognitive control (Anicha et al., 2012; Seli et al., 2015). These observations seem to fit nicely with the reported positive association between Non-Reactivity and memory performances, given the link between cognitive control and memory encoding (Chiu et al., 2015a; b; Krebs et al., 2013; Rosner et al., 2015; Sperduti et al., 2017b). The explanation of the association between Acting with Awareness and the recollection score associated with lures false recognition is less straightforward. Brown et al. (2016) did not report any association between this facet and memory performances, but they did not investigate false memory. Our result is partially coherent with Yeh and Lu (2017) reporting a positive correlation between dispositional mindfulness as measured by the MASS and the number of recognized critical lures. Even if we employed a different dispositional mindfulness measure, the Acting with Awareness subscale of the FFMQ and the MASS are similar constructs. Indeed, five out of the eight items measuring Acting with Awareness belong to the MASS, and basically, they both assess the tendency to be attentive to the present moment experience. Nevertheless, Yeh and Lu (2017) found this positive association when the items distinctiveness was low, while we found it in a highly distinctive setting. Though, as mentioned earlier, using a virtual environment could lead to cognitive load, impairing encoding processes, it remains to explain why Acting with Awareness should increase false-memory formation. Using verbal material, Dorjee et al. (2015) reported that participants with high, compared to low, trait mindfulness displayed less “semantic cost” (e.g., the difference in the judgement accuracy between unrelated and related pairs) when judging the semantic association of pairs of related and unrelated words. Thus, one hypothesis is that dispositional mindfulness would enhance semantic processes and facilitate the spread of activation in the semantic network during encoding, leading to an increased false-memory formation. We suggest that in a 3-D virtual environment, such semantic propagation still occurs and, despite additional perceptual cues, could lead to phantom recollection, characterized by a recollection of lures due to the strong activation of semantic information during encoding (Brainerd et al., 2003). Nevertheless, the present study does not allow us to bring more evidence to defend this explanation, and further investigations are required to support this statement. Our results on the link between dispositional mindfulness and memory also have methodological implications. They highlight that dispositional mindfulness is a multi-dimensional concept rather than a monolithic process. Moreover, since dispositional mindfulness impacts memory performances, this should systematically be assessed in studies investigating the impact of mindfulness meditation on memory.

## Conclusion

To conclude, our results do not support the claim that mindfulness is associated with an increase in false memory. Indeed, we found that a short mindfulness induction improved memory sensitivity and recollective processes without affecting false-memory rates. Similarly, dispositional mindfulness was mainly associated with better memory performances, except for the Acting with Awareness subscale predicting higher recollection score associated with lures false recognition. The present findings nuance the proposal that mindfulness would affect memory, mostly changing decision-making processes (Levi & Rosentreich, 2019), suggesting a possible effect on both encoding and retrieval processes. Undoubtedly, our study is not conclusive on this question since it is hard to disentangle encoding and retrieval processes only with behavioral measures. Future studies employing neuroimaging techniques (e.g., EEG, fMRI) should shed light on this issue.

The present study presents several limitations. First of all, our sample size is relatively small compared to previous studies. Therefore, a replication of the present study with larger sample size is warranted. Second, we only employed visual material, so we could not directly test the differential impact of mindfulness depending on the studied material on the same participants.

Besides, using a 3-D environment could have induced cognitive load, potentially explaining our failure to replicate previous observations. We are already running a follow-up study to investigate these issues. Finally, a limitation shared with most of the studies on the impact of mindfulness meditation on memory is that we employed a short meditation session (15 min). Thus, it could be that this short duration is not enough to produce a measurable effect on false memory. Accordingly, some previous studies also did not find any effect on cognition after a short meditation session (Droit-Volet et al., 2015; Girardeau et al., 2020; Johnson et al., 2015; Larson et al., 2013). Nevertheless, we did find an effect on memory sensitivity, and the cognitive state reported by our participants after the mindfulness induction was in the expected direction. Thus, it is not likely that our results are due to a lack of effectiveness of our experimental manipulation. Another limitation is that the manipulation check was executed after the induction and based only on self-report. Further studies employing more extensive meditation practice and other mindfulness state assessments are required, as suggested by recent critics targeting the research field of mindfulness (Davidson & Kaszniak, 2015).

To our knowledge, this is the first study investigating the impact of mindfulness on memory formation in a complex environment closer to real-life. Our results show that state and dispositional mindfulness could have an overall beneficial effect on memory and encourage employing these practices as a stimulation or remediation tool against memory impairments.

## Supplementary Information

Below is the link to the electronic supplementary material.

Supplementary file1 (DOCX 17 KB)
