# Supplementary material for: Mindfulness and false memories: state and dispositional mindfulness does not increase false memories for naturalistic scenes presented in a virtual environment
Source: Psychol Res. 2021 Apr 13;86(2):571–84. doi: 10.1007/s00426-021-01504-7 (PMC8885469; doi:10.1007/s00426-021-01504-7)
Supplement: Supplementary file 1 — Supplementary file1 (DOCX 17 KB) [file 426_2021_1504_MOESM1_ESM.docx]

**Table 4:** Descriptive statistics and between groups comparison of the manipulation checks

|  | ***Mindfulness*** | ***Story*** | ***p value*** |
| --- | --- | --- | --- |
| Sleepiness | 64.10 (21.71) | 40.56 (25.39) | 0.002** |
| External absorption | 37.89 (23.23) | 24.77 (20.50) | 0.05* |
| Internal absorption | 46.55 (26.96) | 38.71 (31.47) | 0.38 |
| Body absorption | 63.26 (22.75) | 22.18 (23.50) | < 0.001*** |
| Mind Wandering | 56.61 (28.98) | 46.12 (33.92) | 0.28 |
| Mind Focus | 51.32 (28.41) | 23.40 (22.71) | < 0.001*** |

Between parentheses is reported the standard deviation.

**Table 5:** Post-hoc analyses for false recognition with multiple comparisons between items

|  | ***Neutral*** | ***Perceptual*** | ***Semantical*** |
| --- | --- | --- | --- |
| Perceptual | < 0.01 | - | - |
| Semantical | < 0.01 | 0.3 | - |
| Critical | < 0.01 | < 0.01 | < 0.01 |

**Table 6:** Post-hoc analyses for A’ with multiple comparisons between items

|  | ***Neutral*** | ***Perceptual*** | ***Semantical*** |
| --- | --- | --- | --- |
| Perceptual | < 0.01 | - | - |
| Semantical | < 0.01 | 0.8 | - |
| Critical | < 0.01 | < 0.01 | < 0.01 |

**Table 7:** Post-hoc analyses for response bias with multiple comparisons between items

|  | ***Neutral*** | ***Perceptual*** | ***Semantical*** |
| --- | --- | --- | --- |
| Perceptual | < 0.01 | - | - |
| Semantical | < 0.01 | 0.18 | - |
| Critical | < 0.01 | 0.01 | < 0.01 |

**Table 8:** Post-hoc analyses for lures recollection with multiple comparisons between items

|  | ***Neutral*** | ***Perceptual*** | ***Semantical*** |
| --- | --- | --- | --- |
| Perceptual | < 0.01 | - | - |
| Semantical | 0.05 | 0.18 | - |
| Critical | < 0.01 | 0.01 | < 0.01 |

**Table 9:** Post-hoc analyses for false familiarity with multiple comparisons between items

|  | ***Neutral*** | ***Perceptual*** | ***Semantical*** |
| --- | --- | --- | --- |
| Perceptual | 0.05 | - | - |
| Semantical | 0.05 | 1.00 | - |
| Critical | < 0.01 | 0.02 | 0.02 |
